# *O*-GlcNAcylation of RIPK1 rescues red blood cells from necroptosis

**DOI:** 10.3389/fimmu.2023.1160490

**Published:** 2023-06-09

**Authors:** Junghwa Seo, Yeolhoe Kim, Suena Ji, Han Byeol Kim, Hyeryeon Jung, Eugene C. Yi, Yong-ho Lee, Injae Shin, Won Ho Yang, Jin Won Cho

**Affiliations:** ^1^ Glycosylation Network Research Center, Yonsei University, Seoul, Republic of Korea; ^2^ Department of Systems Biology, College of Life Science and Biotechnology, Yonsei University, Seoul, Republic of Korea; ^3^ Department of Molecular Medicine and Biopharmaceutical Sciences, School of Convergence Science and Technology and College of Medicine or College of Pharmacy, Seoul National University, Seoul, Republic of Korea; ^4^ Department of Internal Medicine, Yonsei University College of Medicine, Seoul, Republic of Korea; ^5^ Department of Chemistry, Yonsei University, Seoul, Republic of Korea

**Keywords:** necroptosis, receptor interacting protein kinase1 (RIPK1), *O*-GlcNAcylation, erythrocyte, red blood cell

## Abstract

Necroptosis is a type of cell death with excessive inflammation and organ damage in various human diseases. Although abnormal necroptosis is common in patients with neurodegenerative, cardiovascular, and infectious diseases, the mechanisms by which *O*-GlcNAcylation contributes to the regulation of necroptotic cell death are poorly understood. In this study, we reveal that *O*-GlcNAcylation of RIPK1 (receptor-interacting protein kinase1) was decreased in erythrocytes of the mouse injected with lipopolysaccharide, resulting in the acceleration of erythrocyte necroptosis through increased formation of RIPK1-RIPK3 complex. Mechanistically, we discovered that *O*-GlcNAcylation of RIPK1 at serine 331 in human (corresponding to serine 332 in mouse) inhibits phosphorylation of RIPK1 at serine 166, which is necessary for the necroptotic activity of RIPK1 and suppresses the formation of the RIPK1-RIPK3 complex in *Ripk1* ^-/-^ MEFs. Thus, our study demonstrates that RIPK1 *O*-GlcNAcylation serves as a checkpoint to suppress necroptotic signaling in erythrocytes.

## 1 Introduction

Erythrocytes are the most abundant cell type in the human body and a central player in the transfer of oxygen (1). They serve as the primary or secondary target of pathogenic infections; thus the disruption of erythrocytes integrity during infections, such as sepsis and malaria anemia, accelerates their clearance by macrophages (2, 3). Features that contributed to the loss of erythrocyte integrity and result in the abnormal function of erythrocytes include diminished erythrocyte deformability, a sharp reduction in hemoglobin concentration, and decreased membrane stiffness (1, 4). In addition, the production of erythrocytes decreases as a result of a systemic inflammatory response (5). Therefore, a better understanding of erythrocyte cell death regulation is needed to improve the maintenance of erythrocytes in human diseases.

Erythrocytes function as immunomodulators by binding to inflammatory mediators, which include more than 40 cytokines and nucleic acids, to regulate the activity and maturation of immune cells (6, 7). For example, erythrocytes contribute to the maintenance of immune homeostasis through the *in vivo* scavenging of mitochondrial DNA, which increases after necroptosis (a programmed form of necrosis, or inflammatory cell death) (6, 8). Necroptosis is a type of cell death with excessive inflammation and organ damage in a variety of diseases involving inflammatory processes, including sepsis, inflammatory bowel disease, and neurodegenerative diseases, and thus contributes to the innate immune response during infectious diseases (9). Necroptosis is induced by the activation of distinct cellular receptors, including the tumor necrosis receptor (TNFR1) and toll-like receptors (TLR4 and TLR3) (10). During necroptotic cell death, the formation of a receptor-interacting protein kinases 1 and 3 (RIPK1 and RIPK3, respectively) complex induces the phosphorylation of pseudo kinase mixed lineage kinase domain-like (MLKL), which destroys cell membranes (11–14). In erythrocytes, RIPK1–RIPK3 complex formation and necroptosis by bacterial toxins occur in a manner similar to that of nucleated cell necroptosis (15).

Recently, it was reported that *O*-GlcNAcylation of RIPK3 suppresses necroptosis by inhibiting its interaction with RIPK1 in two septic inflammation models (endotoxin shock induced by intraperitoneal [i.p.] lipopolysaccharide (LPS) injection and the cecal ligation and puncture [CLP] procedure) and in a mouse model of Alzheimer’s disease (16, 17). *O*-GlcNAcylation is one of the most sensitive and dynamic posttranslational modifications (PTMs), which is catalyzed by *O*-GlcNAc transferase (OGT) and eliminated by *O*-GlcNAcase (OGA), and modulates a myriad of cellular events, including the host immune response and signal transduction during pathogen infection (18–20). Since mature erythrocytes have been enucleated, changes in PTMs of pre-existing proteins rather than the synthesis of new genes would regulate cell death signaling. Moreover, changes in PTMs in infected erythrocytes are also dynamic (21, 22). Erythrocytes are readily available from blood samples. Under conditions alternating cellular *O*-GlcNAcylation, changes in the PTM of pre-existing proteins rather than the expression of new proteins act as variables, making it easy to interpret the experimental results. The *O*-GlcNAc cycling enzymes, OGT and OGA, are present in erythrocytes and multiple erythrocyte proteins are modified by *O*-GlcNAc (23). Despite the aforementioned advances in knowledge, how erythrocytes utilize *O*-GlcNAc cycling to control necroptosis signaling remains unclear.

This study demonstrated that RIPK1, a necessary molecule in the progression to necroptosis, is a novel substrate for OGT in erythrocytes. We found that *O*-GlcNAcylation of RIPK1 was decreased during LPS-induced endotoxemia and a reduction of RIPK1 *O*-GlcNAcylation was blocked by Thiamet-G (TMG), an OGA inhibitor. TMG administration renders diminishment of necroptosis in erythrocytes along with a decrease in the phosphorylation of serine 166 in RIPK1. In addition, TMG administration interfered with the formation of the RIPK1–RIPK3 complex and protected the pathological effect on erythrocytes induced by LPS-induced endotoxemia. To further investigate the molecular mechanism shown in erythrocytes, we used Mouse Embryonic Fibroblast (MEF) *Ripk1* ^-/-^ cells because there were limitations in testing the necroptosis modulating effect of *O*-GlcNAcylation through genetic engineering of mature erythrocytes. Mechanistically, we discovered that the *O*-GlcNAcylation of serine 331 on RIPK1 in human (corresponding to serine 332 in mouse) is particularly responsible for regulating necroptosis in MEF *Ripk1* ^-/-^ cells reconstituted with RIPK1. Taken together, we demonstrated that *O*-GlcNAcylation of RIPK1 on serine 331 in human (corresponding to serine 332 in mouse) modulates necroptosis and these findings suggest that *O*-GlcNAcylation plays an important role in the fine-tuning of necroptosis in erythrocytes.

## 2 Results

### 2.1 Reduction of *O*-GlcNAcylation modulates damage to erythrocytes during LPS-induced endotoxemia

**Figure 1:**
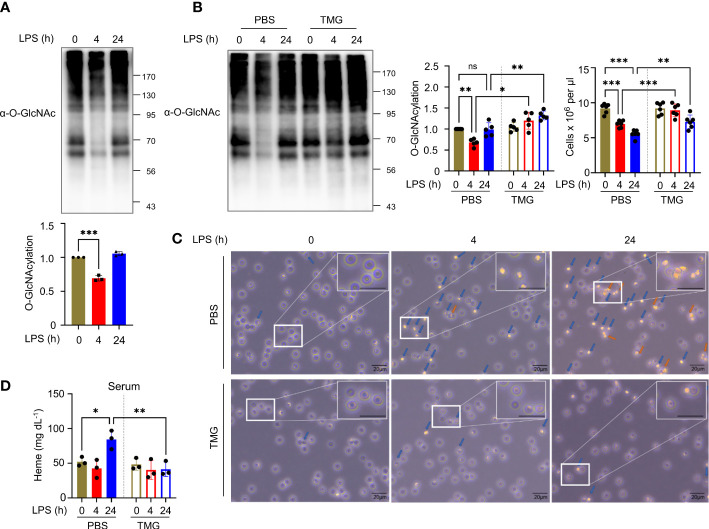
Reduced *O*-GlcNAcylation of erythrocytes in LPS-injected mice is involved in erythrocyte destruction. **(A)** Western blot analysis showing the levels of *O*-GlcNAcylation in erythrocytes at various times after i.p. injection with 10 mg kg^-1^ day^-1^ LPS (lipopolysaccharide from *Escherichia coli* O111:B4). Cellular *O*-GlcNAcylation was normalized to GAPDH. (n=3) **(B)** Western blots showing the levels of *O*-GlcNAcylation in erythrocytes at the indicated times after i.p. injection with 10 mg kg^-1^ day^-1^ LPS and 40 mg kg^-1^ day^-1^ Thiamet-G (TMG) or the same volume of PBS (as a control). In the left graph, cellular *O*-GlcNAcylation was normalized to GAPDH. In the right graph, the number of erythrocytes isolated from whole blood obtained from the indicated groups of mice is shown by cell counting analyses. (n=5) **(C)** Light microscopy (LM) showing changes in the shape of erythrocytes. Before LM observations, erythrocytes obtained from LPS-injected mice (10 mg kg^-1^ day^-1^) with TMG (40 mg kg^-1^ day^-1^) or PBS (as a control) administration was incubated in RPMI-1640 for 30 min. blue arrows; abnormal morphologies, orange arrows; aggregate. The enlarged area was selected from the original at 400x magnification. Scale bar, 20 μm. **(D)** Heme assay to measure the concentration of free heme released into the serum. Serum was harvested at the indicated times after i.p. injection with 10 mg kg^-1^ day^-1^ LPS and 40 mg kg^-1^ day^-1^ TMG or the same volume of PBS (as a control). (n=3) (**A**, **B**, **D**; one-way ANOVA with Sidak’s multiple comparisons test). Data are presented as the mean ± standard deviation (SD); statistical significance was annotated as *P < 0.05; **P < 0.01; ***P < 0.001.

LPS, a Gram-negative bacterial endotoxin, injection alone without caspase inhibition induces RIPK1/RIPK3-mediated necroptosis in various organs and systemic inflammation *in vivo* (24). LPS is recognized by TLR4 expressed on the surface of several immune cells, including macrophages, dendritic cells, and several T cell populations, but its expression in non-immune cells is less documented (25). In a model of septic inflammation, endotoxin shock induced by intraperitoneal LPS injection results in the progression of necroptosis and secretion of inflammatory cytokines in macrophages (16). In addition, LPS attenuates the hexosamine biosynthesis pathway (HBP) (26) and protein *O*-GlcNAcylation in macrophages (16). Several studies have indicated that the administration of TMG or NButGT (27), an OGA inhibitor, improves the survival of LPS-injected mice (28, 29) and has a protective effect on cardiovascular function or lung-tissue damage, which suggests that *O*-GlcNAcylation is deeply engaged in LPS-induced pathophysiological changes (30, 31). However, the role of *O*-GlcNAcylation in the functional disruption of erythrocytes induced by LPS-induced endotoxemia (32, 33) should be further investigated. Using erythrocytes isolated from the whole blood of LPS i.p. injected mice, we evaluate changes in erythrocyte *O*-GlcNAcylation. We observed that total *O*-GlcNAcylation in erythrocytes was attenuated by LPS at 4h and it was restored at 24h (**Figures 1A**, **S1A**). As a reduction in *O*-GlcNAcylation was observed at 4h after injection, we increased the level of *O*-GlcNAcylation in the erythrocytes by administering TMG (**Figures 1B**, **S1B**). The results indicated that the diminishment of erythrocyte cell number in LPS-induced endotoxemia was ameliorated compared with LPS injection only (**Figure 1B**). The *O*-GlcNAcylation levels in erythrocytes were restored at 24h, but OGA activity after LPS injection was still higher compared to 0h (**Figure S1C**). Next, the morphological destruction of erythrocytes by LPS (1) was also prevented by TMG administration (**Figure 1C**). We confirmed that the level of free heme released by erythrocytes into serum in response to LPS was also suppressed by TMG administration (**Figure 1D**). Therefore, these results indicate that preventing a decrease in *O*-GlcNAcylation can ameliorate erythrocyte damage during LPS-induced endotoxemia. Furthermore, We investigated the effect of TMG administration on LPS-induced lethality. An improvement in survival rate was observed in mice that were administered TMG than in LPS only–injected mice (**Figure S1D**). Several potential *O*-GlcNAcylated target proteins (29), including previously reported RIPK3 (16), could be affected by *O*-GlcNAcylation in LPS–injected conditions. Our results support that TMG has protective effects during LPS injection through an elevation of *O*-GlcNAcylation.

### 2.2 RIPK1 *O*-GlcNAcylation inhibits its interaction with RIPK3 in erythrocytes

**Figure 2:**
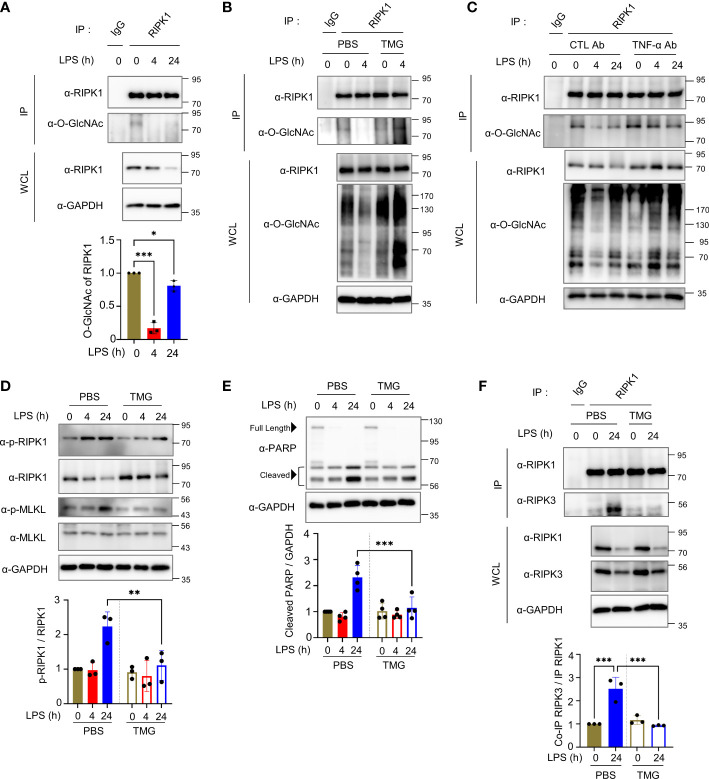
Decreased *O*-GlcNAcylation of endogenous RIPK1 in mouse erythrocytes during LPS injection enhances its interaction with RIPK3 to promote necroptotic cell death. **(A)** Top: immunoprecipitation (IP) assay to determine *O*-GlcNAcylation of endogenous RIPK1 in erythrocytes isolated from LPS (10 mg kg^-1^ day^-1^)-injected mice during the indicated times. Bottom: in the graph, RIPK1 O-GlcNAcylation was normalized to immunoprecipitated RIPK1. In panels **(A–C, F)**, the same amount of RIPK1 protein was immunoprecipitated from whole erythrocyte cell lysates. (n=3) **(B)** IP assay showing the effect of TMG (40 mg kg^-1^ day^-1^) administration on *O*-GlcNAcylation levels of RIPK1 in erythrocytes. **(C)** IP assay showing the effect of TNF-α antibody (10 mg kg^-1^ day^-1^) in LPS-injected mice on *O*-GlcNAcylation levels of RIPK1 in erythrocytes. To neutralize TNF-α, TNF-α antibody (10 mg kg^-1^) was i.p. injected 4 hours before LPS injection. **(D, E)** Top: Western blots showing the levels of phospho-Ser166-RIPK1 or PARP in response to LPS (10 mg kg^-1^ day^-1^) injection with TMG (40 mg kg^-1^ day^-1^) or PBS (same volume, as a control) over the indicated time in erythrocytes. Bottom: graphs representing the levels of phospho-Ser166-RIPK1 normalized to RIPK1 or cleaved PARP (around 56 kDa) normalized to GAPDH. (**D**: n=3) (**E**: n=4) **(F)** Left: co-IP assay indicating the interaction between RIPK1 and RIPK3 in erythrocytes in 24h LPS (mg kg^-1^ day^-1^) injection. Right: graph explaining the amount of co-immunoprecipitated RIPK3 normalized with immunoprecipitated RIPK1. (n=3) (**A**, **D–F**; one-way ANOVA with Sidak’s multiple comparisons test). Data are presented as the mean ± standard deviation (SD); statistical significance was annotated as *P < 0.05; **P < 0.01; ***P < 0.001.

Although some erythrocyte proteins are regulated by *O*-GlcNAc modification, it remains enigmatic as to how *O*-GlcNAc cycling modulates signal transduction in erythrocytes (23). Tumor necrosis factor-α (TNF-α) is a prime mediator of the inflammatory response and is secreted in large amounts into the bloodstream during LPS injection (34). Little is known about the LPS- or TNF-α-induced cell death signaling pathway in erythrocytes, but erythrocytes have been reported to be reservoirs of various cytokines, including TNF-α (7, 35). Therefore, we examined the *O*-GlcNAcylation of several crucial components of LPS- or TNF-α-induced cell death signaling, which included RIPK1. We found that RIPK1 was a novel *O*-GlcNAcylated protein in erythrocytes, although the previously documented *O*-GlcNAcylation of RIPK3 was not detected (**Figure 2A** and **Figure S2A**, left). We also examined *O*-GlcNAcylation of FADD in erythrocytes, but we were unable to confirm the presence of *O*-GlcNAc modification of FADD, albeit the possibility of *O*-GlcNAcylation of human FADD was raised using proteomics analysis (**Figure S2A**, right) (36). Consistent with previous reports of *O*-GlcNAcylation of several proteins including IKKβ and Caspase-8 (37–39), *O*-GlcNAcylation of IKKβ and Caspase-8 was observed in erythrocyte lysate after immunoprecipitation using the *O*-GlcNAc antibody (**Figures S2C, E**). Also, the *O*-GlcNAc signal of TAK1 was detected in erythrocyte (**Figures S2E, F**) by lectin precipitation using agarose-conjugated succinylated-wheat germ agglutinin beads (20), but the *O*-GlcNAcylation levels of these proteins were not altered by injection of LPS or TNF (**Figures S2C–F**). To investigate whether TNF is indeed regulating *O*-GlcNAcylation of RIPK1, we observed *O*-GlcNAcylation of RIPK1 in erythrocytes after injection of murine TNF-α and found a decrease in *O*-GlcNAcylation of RIPK1, similar to that observed in LPS injection (**Figure S2B**). Notably, *O*-GlcNAcylation of RIPK1 was decreased following LPS injection and the reduction in *O*-GlcNAcylation of RIPK1 by LPS was blocked by TMG (**Figure 2B**). As shown in **Figures 1C, D**, we found that LPS-induced morphological changes and heme release from erythrocytes were suppressed following an LPS-induced decrease in *O*-GlcNAcylation of RIPK1 that was blocked by TMG administration. Next, we examined the *O*-GlcNAcylation levels of RIPK1 after neutralizing TNF-α using TNF-α antibody in LPS-injected mice since LPS-provoked TNF-α secretion was observed in other reports. Importantly, i.p. injection of TNF-α antibody directly prevented the decrease in *O*-GlcNAcylation of RIPK1 induced by LPS in erythrocytes (**Figure 2C**). In addition, it has been observed that the level of free heme released by erythrocytes into the serum in response to LPS is also suppressed by anti-TNF-α (**Figure S2G**). These results suggest that TNF is indeed involved in LPS-injected erythrocyte damage.

The phosphorylation of RIPK1 on serine 166 is critical for necroptosis induction (40, 41). For example, RIPK1 undergoes phosphorylation on serine 166 to promote necroptosis in macrophages (41). Interestingly, erythrocytes also activate the cell death pathway that shares components with necroptosis occurring in nucleated cells by bacterial toxins (15). Therefore, to elucidate the molecular mechanism through which the reduced *O*-GlcNAcylation of RIPK1 during LPS-induced endotoxemia results in erythrocyte damage, we determined whether serine 166 of RIPK1 is also phosphorylated in erythrocytes. We observed that phospho-Ser166-RIPK1 was elevated by LPS injection; however, phospho-Ser166-RIPK1 levels were attenuated by TMG administration compared with LPS injection alone (**Figure 2D**). In addition, an increase in cleaved PARP (around ~55 kDa), which was reported as a necroptosis product (42), was inhibited by TMG administration (**Figure 2E**). However, administration of TMG did not significantly affect the amount or cleavage of full-length caspase-3, 6, 7, and 8 in mice injected with LPS (**Figure S2H**). As shown in **Figure 1B**, *O*-GlcNAcylation levels in erythrocytes were restored at 24h when necroptosis was highly activated. Nevertheless, when the reduction of *O*-GlcNAcylation, which appears as an early response, was blocked through TMG, the cell death signal of erythrocytes was continuously suppressed for up to 24h. Therefore, we focused on the formation of the RIPK1–RIPK3 complex to promote necroptosis signaling and found that the interaction between RIPK1 and RIPK3 was significantly antagonized as a result of the restoration in *O*-GlcNAcylation of RIPK1 by TMG administration (**Figures 2F**, **S2I**).

### 2.3 *O*-GlcNAcylation regulates TSZ-induced necroptosis by modulating phospho-Ser166-RIPK1

**Figure 3:**
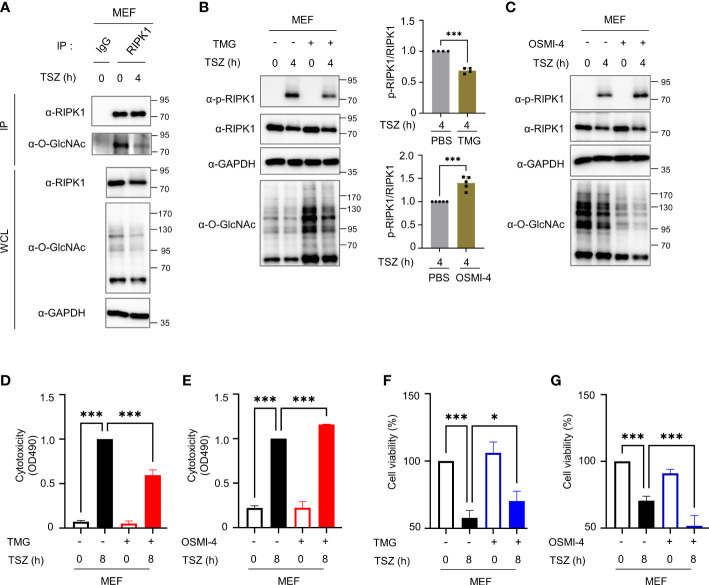
*O*-GlcNAcylation modulates TSZ-induced phosphorylation of RIPK1(Ser166) and necroptotic cell death in mouse embryonic fibroblasts (MEFs). **(A)** Immunoprecipitation (IP) assay showing the *O*-GlcNAcylation of endogenous RIPK1 in MEFs in response to TSZ (TSZ; combinations of chemicals, 40 ng ml^-1^ of mouse TNF-α + 20 mM Smac mimetic compound + 30 μM z–VAD–FMK). **(B, C)** Western blots showing the levels of phospho-Ser166-RIPK1 following treatment with TSZ for 4h after pretreatment with 1 μM TMG **(B)** or 2 μM OSMI-4 **(C)** in MEFs. Top **(B)**, Bottom **(C)**: graphs showing the amount of phospho-Ser166-RIPK1 normalized to RIPK1 on (**B**: n=4) (**C**: n=5) **(D, E)** LDH release assay to reflect the cytotoxicity of MEFs in necroptosis via TSZ stimulation; 1 μM TMG **(D)** or 2 μM OSMI-4 **(E)** were pretreated before an 8h TSZ stimulation. (**D**: n=4) (**E**: n=3) **(F, G)** CellTiter-Glo assay to measure the cell viability of MEFs in necroptosis following TSZ stimulation. 1 μM TMG **(F)** or 2 μM OSMI-4 **(G)** were pretreated before an 8h TSZ stimulation. The y-axis starts at 50%. (**F**: n=4) (**G**: n=4) (**B**, **C**; Unpaired two-tailed *t*-test). (**D–G**; one-way ANOVA with Sidak’s multiple comparisons test). Data are presented as the mean ± standard deviation (SD); statistical significance was annotated as *P< 0.05; ***P < 0.001.

Based on our results, we further investigated whether *O*-GlcNAcylation is directly involved in the progression of necroptosis. We failed to induce necroptosis following LPS- or TNF-α treatment of erythrocytes obtained from mice *in vitro* (data not shown), we induced necroptosis by treating MEFs with TSZ (TNF in combination with a Smac–mimetic compound and the pan-caspase inhibitor Z–VAD–FMK) (43, 44), which is reported to be more suitable for inducing necroptosis than LPS+Z–VAD (45). We confirmed that cellular *O*-GlcNAcylation levels and *O*-GlcNAcylation of RIPK1 were attenuated by TSZ treatment, as was the case when necroptosis was induced in erythrocytes by *in vivo* LPS-induced endotoxemia (**Figures 3A**, **S3A**). Thereafter, we investigated whether the inhibition of necroptosis caused by TMG in erythrocytes observed in **Figure 2** was also confirmed in MEFs. Although MEFs cannot fully reflect erythrocytes because erythrocytes are enucleated cells in a different niche than MEFs, cellular *O*-GlcNAcylation reduced by TSZ was restored with TMG pretreatment and it reduced the level of phospho-Ser166-RIPK1 (**Figure 3B**). Conversely when pretreatment with OSMI-4 was performed to inhibit OGT activity, TSZ-induced phospho-Ser166-RIPK1 was further augmented (**Figure 3C**). To further investigate whether *O*-GlcNAcylation of RIPK1 affects its protein stability, we measured RIPK1 protein levels in HT-29 or HEK293 cell lines with TMG or OGT knockdown and found no significant changes in RIPK1 protein levels (**Figure S3B**). This observation is consistent with **Figures 3B, C**, which showed that RIPK1 protein levels were not affected by TMG or OSMI-4 treatment in MEF cells. Next, we performed an LDH release assay to compare the cytotoxicity of TSZ following pretreatment with TMG or OSMI-4, and these results were accompanied by corresponding changes in phospho-Ser166-RIPK1 levels (**Figures 3D, E**). The decrease in cell viability by TSZ was recovered by TMG, but OSMI-4 further accelerated necroptosis (**Figures 3F, G** and **Figure S3C, D**).

### 2.4 RIPK1 is modified with *O*-GlcNAc on serine 331

**Figure 4:**
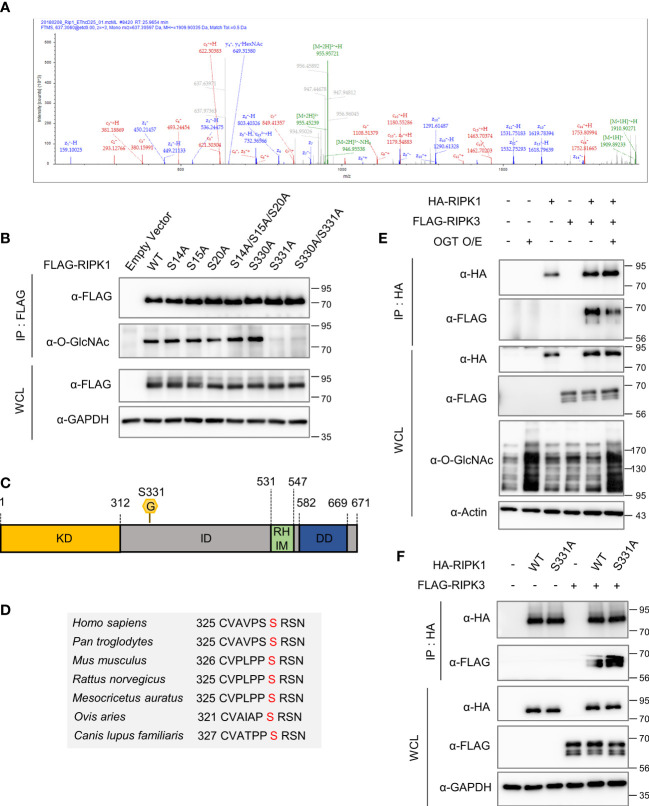
Serine 331 of RIPK1 is a major *O*-GlcNAcylation site and inhibits its interaction with RIPK3. **(A)** Mass spectrometry (MS) analysis identifies the *O*-GlcNAcylation sites in RIPK1. EThcD spectra of O-Glycopeptide CVAVPSSRSNSAT from human RIPK1 are shown. The site of *O*-GlcNAc modification was identified as serine 331. The c and z fragments detected are as indicated in the sequence. **(B)** IP assay to compare the *O*-GlcNAcylation levels of wild-type RIPK1 with S to A mutant candidates. 10 μg of either FLAG-RIPK1 WT or mutants were transfected into HEK293 cells and lysed 24h later. **(C)** Human RIPK1 protein structure. *O*-GlcNAcylation was indicated at serine 331 located in the intermediate domain. (KD, kinase domain; ID, intermediated domain; RHIM, RIP homotypic interaction motif domain; DD, death domain) **(D)** Sequence information indicating that RIPK1 serine 331 is a highly conserved sequence in mammals. **(E)** Co-IP assay evaluating the interaction between overexpressed RIPK1 and RIPK3 in HEK293 cells. 5 μg of non-tagged OGT was co-transfected with 10 μg of HA-RIPK1, FLAG-RIPK3, or empty vector (-). **(F)** Co-IP assay evaluating the interaction between overexpressed RIPK1 WT or S331A and RIPK3 in HEK293 cells. 10 μg of HA-RIPK1 or FLAG-RIPK3 were overexpressed and lysed 24h later.

To detect the major *O*-GlcNAcylation site(s) on RIPK1, we performed mass spectrometry (MS) analysis using HEK293 cells, which is facile for DNA overexpression. Using MS analysis, we identified *O*-GlcNAcylation sites of hRIPK1at S14, S15, S20, S330 and S331 (**Figures 4A**, **S4A–D**). After checking whether the experimental system worked well by confirming that FLAG-RIPK3, as well as, FLAG-RIPK1 were *O*-GlcNAcylated in HEK293 (data not shown), we generated several point mutants in which Ser was substituted with Ala using the five *O*-GlcNAcylation sites that we analyzed. Of these residues, we found that *O*-GlcNAcylation was drastically abolished in the S331A mutant compared with the wild-type (WT) (**Figure 4B**). Serine 331 is a highly species-conserved site in mammals, located within the intermediate domain of RIPK1 (**Figures 4C, D**). To examine whether the *O*-GlcNAcylation levels of mouse S332 corresponding to human S331 are indeed declined compared to WT, we overexpressed RIPK1 WT or RIPK1 S332A in MEF ripk1^-/-^ cells. As expected, *O*-GlcNAcylation levels were significantly reduced in S332A compared to WT, and unlike WT, *O*-GlcNAcylation levels did not show changes in response to necroptosis stimulation treated with TSZ (**Figure S4E**). Although the *O*-GlcNAcylation site of RIPK1 in erythrocytes could not be tested through M/S analysis, these results may suggest that the *O*-GlcNAcylation site identified in HEK293 through M/S analysis is conserved in mice. Next, we confirmed that the interaction between overexpressed RIPK1 and RIPK3 was also inhibited by increased *O*-GlcNAcylation through TMG administration, as in erythrocytes. Consistent with previous reports that *O*-GlcNAcylation on Thr467-RIPK3 inhibits its interaction with RIPK1 (16), the amount of the RIPK1–RIPK3 complex decreased following OGT overexpression or TMG treatment (**Figures 4E**, **Figure S4F**). Combining these results with TMG-induced inhibition of RIPK1-RIPK3 complex formation in erythrocytes (**Figure 2F**), we raised the possibility that *O*-GlcNAcylation, in addition to the already known RIPK3 *O*-GlcNAcylation, would bidirectionally regulate RIPK1-RIPK3 complex formation via RIPK1. Notably, the binding between RIPK1 S331A and RIPK3 was strengthened compared with that of RIPK1 WT, suggesting that not only RIPK3 *O*-GlcNAcylation but also RIPK1 serine 331 *O*-GlcNAcylation regulates the interaction between RIPK1 and RIPK3, and thereby tightly regulates necroptosis (**Figure 4F**).

### 2.5 *O*-GlcNAcylation of RIPK1 serine 331 interferes with TSZ-induced necroptosis by inhibiting phospho-Ser166-RIPK1

**Figure 5:**
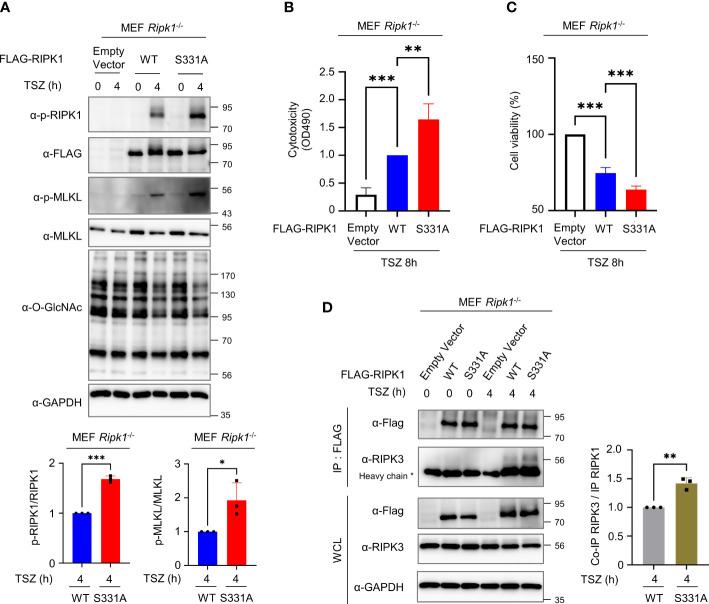
*O*-GlcNAcylation of RIPK1 serine 331 inhibits necroptosis induced by TSZ treatment in MEF *Ripk1* ^-/-^ cells. **(A)** Top: Western blots showing the levels of phospho-Ser166-RIPK1 and phospho-Ser345-MLKL. Bottom: graphs showing the amount of phospho-Ser166-RIPK1 normalized to RIPK1 (left) or phospho-Ser345-MLKL normalized to MLKL (right). Before a 4h TSZ stimulation, 10 μg of RIPK1 WT or S331A DNA construct was transfected into MEF *Ripk1* ^-/-^ cells. (**A**-left: n=3) (**A**-right: n=3) **(B)** LDH release assay reflecting the cytotoxicity of MEF *Ripk1* ^-/-^ cells in necroptosis following an 8h TSZ stimulation. Before stimulation, 10 μg of each DNA construct was transfected. (n=3) **(C)** CellTiter-Glo assay to measure the cell viability of MEF *Ripk1* ^-/-^ cells in necroptosis following TSZ stimulation. Before an 8h TSZ stimulation, 10 μg of each DNA construct was transfected. The y-axis starts at 50%. (n=4) **(D)** Co-IP assay to evaluate the interactions between RIPK1 WT or S331A and endogenous RIPK3 in MEF *Ripk1* ^-/-^ cells. 10 μg of each DNA was overexpressed for 20–24h before TSZ treatment. After a 4h TSZ stimulation, cells were lysed and analyzed to detect the indicated proteins. (n=3) (**A**-left, **A**-right**, D**; Unpaired two-tailed *t*-test). (**B**, **C**; one-way ANOVA with Sidak’s multiple comparisons test). Data are presented as the mean ± standard deviation (SD); statistical significance was annotated as *P < 0.05; **P < 0.01; ***P < 0.001.

**Figure 6:**
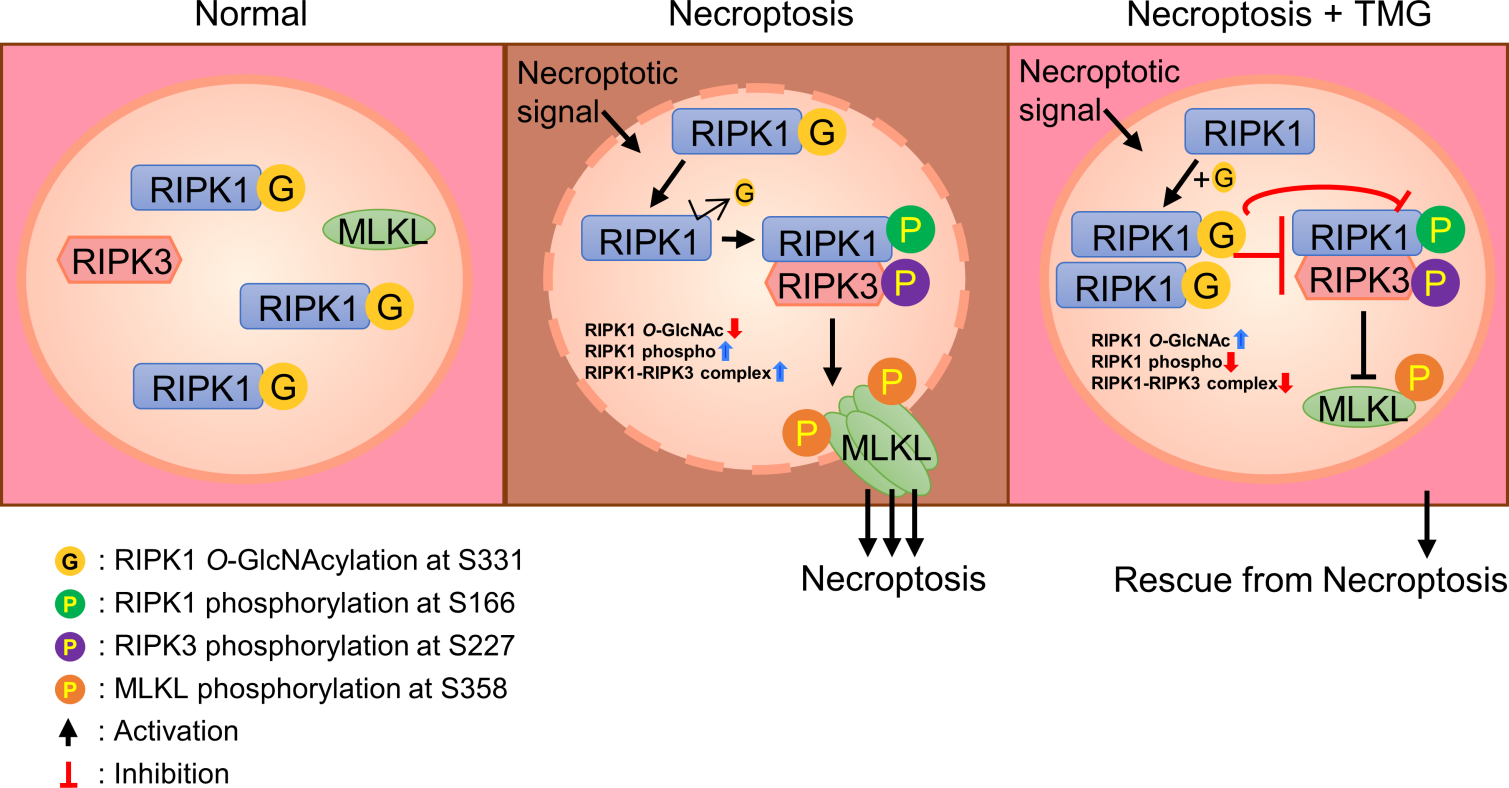
A schematic diagram describing the predicted function of RIPK1 *O*-GlcNAcylation in erythrocytes. In the absence of a necroptotic signal, *O*-GlcNAcylation of serine 331 in human erythrocytes (serine 332 in mouse erythrocytes) occurs. After stimulation of erythrocytes necroptosis signaling, *O*-GlcNAcylation of RIPK1 is reduced. As a result of reduced *O*-GlcNAcylation of RIPK1, phospho-Ser-166 of RIPK1 is enhanced and the formation of a RIPK1–RIPK3 complex is facilitated. These processes promote the phosphorylation of MLKL and enhance erythrocyte necroptosis. TMG restores the reduced *O*-GlcNAcylation of RIPK1, blocking phospho-Ser-166 of RIPK1 and inhibiting the formation of a RIPK1–RIPK3 complex. An increase in RIPK1 *O*-GlcNAcylation inhibits necroptosis of erythrocytes.

To determine whether *O*-GlcNAcylation of RIPK1 Ser331 is directly responsible for inhibiting necroptosis, we reconstituted RIPK1 WT or S331A in MEFs *Ripk1* ^-/-^ and induced necroptosis by TSZ treatment. As a result, we confirmed that S331A exhibited higher levels of both p-RIPK1 and p-MLKL, specific markers of necroptosis, compared with the WT (**Figure 5A**). Then, we measured LDH release and cell viability of WT and S331A to compare the function of WT RIPK1 or S331A RIPK1 during a TSZ-induced necroptosis signal. As expected, S331A showed higher cytotoxicity and lower cell viability than the WT (**Figures 5B, C**, **S5A**). To clarify whether *O*-GlcNAcylation of serine 331 specifically regulates RIPK1 activity in necroptosis, we induced apoptosis by TS treatment without z-VAD. Unlike S331A in necroptosis, there was no difference in cytotoxicity compared to WT in our TNF-induced apoptosis (**Figure S5B**). Importantly, RIPK1 S331A, in which the *O*-GlcNAc modification site is mutated, interacted better with RIPK3 under TSZ treatment than with the WT (**Figure 5D**). Taken together, these results support our hypothesis that *O*-GlcNAcylation of RIPK1 at serine 331 is a major and novel posttranslational modification that interferes with necroptosis signaling. Because of technical difficulties in performing DNA transfection in erythrocytes, we could not directly test the function of *O*-GlcNAcylation at serine 331 through overexpression of RIPK1 S331A. However, we demonstrated that *O*-GlcNAcylation of RIPK1 inhibits the necroptotic activity of RIPK1 in erythrocytes and RIPK1-reconstituted MEFs *Ripk1* ^-/-^ after stimulating necroptosis. Based on the results regarding the mutation of Ser331-RIPK1 *O*-GlcNAcylation, we developed a schematic depicting describing the predicted role of *O*-GlcNAcylation on RIPK1 in erythrocytes (**Figure 6**).

## 3 Discussion

Erythrocytes are the most abundant cell type in the human body and function as an immunomodulator as well as an oxygen supply. Nevertheless, the molecular mechanism of erythrocyte necroptosis is poorly understood. In this study, we identified the mechanism by which *O*-GlcNAcylation of serine 331 of RIPK1 inhibits phosphorylation of RIPK1 on serine 166, concurrent interferes with the RIPK3 interaction and thus suppresses necroptosis. Together, our results suggest that *O*-GlcNAcylation is a major PTM that regulates erythrocytes necroptosis as well as erythropoiesis (46).

In addition, RIPK1 is emerging as a therapeutic target in Alzheimer’s and Parkinson’s disease, in which irregular neuronal cell death occurs (47, 48). For the treatment of diseases in which dysregulation of necroptosis occurs, such as neurodegenerative diseases, sepsis, and cancer, RIPK1 serine 331 *O*-GlcNAcylation warrants further study as a useful therapeutic target or strategy.

Although *O*-GlcNAcylation governs diverse functions in various cells, identifying *O*-GlcNAcylated proteins in erythrocytes and how they utilize *O*-GlcNAc to control their own fate in response to external stimuli has not been under the spotlight. In this study, we suggest a molecular mechanism for erythrocyte necroptosis by LPS-induced endotoxemia, by elucidating that RIPK1 *O*-GlcNAcylation suppresses necroptosis signaling in erythrocytes. *O*-GlcNAcylation may be engaged in sepsis-induced erythrocyte necroptosis. Expanding on this, we propose that the identification of additional *O*-GlcNAcylated targets in erythrocytes will lead to new biomarkers for abnormal cell survival/death-related diseases.

We observed an increased survival rate following TMG administration in LPS-induced pathogenesis (**Figure S1F**). In addition, previous studies demonstrated that increasing *O*-GlcNAcylation through NButGT, TMG, or glucosamine intake protects the cardiovascular system and lung tissue from damage resulting from sepsis (28–31). Since LPS injection can replicate many physiological characteristics of sepsis (49), additional mechanistic and translational research of TMG for clinical application is required.

In this study, we focused on the role of *O*-GlcNAcylation in erythrocytes, which are cells that are not specialized in the immune response, but modulate the immune response by recruiting or activating other immune cells, such as macrophages and neutrophils. It is noteworthy that mature human and murine erythrocytes, which are considered to have only shallow functions, are bona fide conductors of immune signaling. Since we failed to stimulate erythrocytes isolated from mice by LPS or TNF treatment *in vitro* (data not shown), it is not possible to clearly discern which signal transduction in erythrocytes is responsible for necroptosis. However, we have shown that necroptosis occurs in erythrocytes as a result of a systemic response to LPS-induced endotoxemia *in vivo*. Necroptosis can be triggered by various signals, including TNF, CD95L, TRAIL, PAMPs, LPS, and IFNs (50). TLR4 activation triggers downstream signaling pathways, leading to the production and release of various cytokines and death ligands. LPS induces the activation of monocytes and macrophages and then activated macrophages produce inflammatory cytokines including TNF-α, IL-6, and IL-12 (51). Therefore, massive cytokine secretion into serum in the LPS-induced endotoxemia model is supported by previous studies (52). However, analysis of TLR4 expression and LPS-TLR4 signaling transduction in erythrocytes is not well-established. Among the triggers of necroptosis, TNF receptors exist in the erythrocytes membrane and the TNF-α concentrations in erythrocytes are 10-fold higher than in plasma (4, 7). Based on these findings, we speculate necroptosis in erythrocytes is induced by TNF-α under systemic infection conditions implemented by LPS injection, although whether this TNF-α directly contributed to the induction of erythrocytes necroptosis is not conclusive. Further investigation is needed to determine whether TNF contributes to necroptosis in erythrocytes, but we observed a reduction in RIPK1 *O*-GlcNAcylation after injecting murine TNF-α, similar to the decrease observed in LPS injection (**Figure S2B**). Furthermore, the decrease in the level of free heme released into the serum by LPS upon neutralization with TNF-α antibody may provide a clue that TNF-α is involved in erythrocyte necroptosis (**Figure S2G**).

The *in vivo* role of RIPK1 has been difficult to assess due to the perinatal mortality of full knockout mice (53). Hematopoietic RIPK1 deficiency results in bone marrow failure through hematopoietic cell death (54). We have technical difficulties in overexpressing the target protein in mature erythrocytes as new gene transcription and protein synthesis are hard due to the lack of a nucleus. Accordingly, the main limitation of our study is that it is ambiguous to explain that the functional mechanism of *O*-GlcNAc-Ser331-RIPK1 also works in erythrocytes. Indeed, it has not yet been determined whether S331A increases necroptosis more than WT RIPK1 in human erythrocytes. Instead, we used MEF *Ripk1* ^-/-^ cells as a necroptotic cell death model to demonstrate a crucial role for RIPK1 serine 331 *O*-GlcNAcylation (**Figure 5**), in which *O*-GlcNAc-Ser331-RIPK1 is primarily responsible for the inhibition of necroptosis. Nevertheless, it should be considered that MEFs do not sufficiently replace erythrocytes biology. Moreover, it is still not known which amino acid(s) in RIPK1 within erythrocytes are responsible for *O*-GlcNAcylation since the M/S analysis used to identify the *O*-GlcNAcylation site of RIPK1 employed HEK293 lysates, not erythrocyte lysates, in this study.

Recently, there have been reports that the *O*-GlcNAcylation of RIPK3, an essential molecule in necroptosis, inhibits necroptotic cell death in macrophages, hepatocytes, and neuronal cells (16, 17, 55). In light of our results, we suggest that *O*-GlcNAcylation of RIPK1 along with the previously reported *O*-GlcNAcylation of RIPK3 is important for antagonizing necroptotic cell death by modulation of the RIPK1–RIPK3 interaction. As we could not detect *O*-GlcNAcylation of RIPK3 in erythrocytes, *O*-GlcNAcylation of RIPK1, rather than RIPK3, appears to be a major regulator of necroptotic signaling at least in erythrocytes. Indeed, the RIPK1 S331A mutant has been shown to enhance LDH release and reduce cell viability during necroptosis following TSZ treatment in MEFs *Ripk1* ^-/-^. Nevertheless, it is important to consider the effect of other posttranslational modifications, such as phosphorylation on serine 331 of RIPK1 (56, 57). However, we found a common reduction in cellular *O*-GlcNAcylation levels in mouse erythrocytes and MEFs in both types of necroptotic signals, LPS and TNF. Moreover, as changes in *O*-GlcNAcylation through TMG or OSMI-4 also regulate necroptosis by modulating phospho-Ser166-RIPK1, our results support that *O*-GlcNAcylation of RIPK1 is a crucial posttranslational modification.

In conclusion, our data show a sophisticated role for *O*-GlcNAcylation on RIPK1 during LPS injection in erythrocytes. Our understanding of the unrecognized immunomodulatory mechanism of *O*-GlcNAcylation during necroptosis may be expanded and harnessed for drug development in necroptosis-associated human diseases.

## 4 Materials and methods

### 4.1 Laboratory animals

Eight-week-old C57BL/6J mice were purchased from DBL (DBL Co., Ltd., Eumseong, South Korea). The Institutional Animal Care and Use Committees of the Laboratory Animal Research Center at Yonsei University approved the experiments (IACUC-A-202107-1296-02).

### 4.2 Cell cultures, plasmids, and transfection

HEK293, MEF wild-type, and MEF *Ripk1* ^-/-^ cells were cultured in Dulbecco’s Modified Eagle’s Medium (Welgene, #LM 001-05, Gyeongsan-si, South Korea) supplemented with 10% fetal bovine serum (Gibco, #16000-044, USA). MEF *Ripk1* ^-/-^ was kindly provided by Dr. You-Sun Kim (Ajou University, Suwon, South Korea). All cell lines were incubated in 5% CO_2_ at 37°C. All cells were tested for mycoplasma contamination using a mycoplasma detection polymerase chain reaction (PCR) test (Cosmogenetech, South Korea). pCMV-7.1-3XFlag-RIPK1 and various point mutant constructs (S14A, S15A, S20A, Triple S->A substitution of S14/S15/S20, S330A, S331A, double S->A substitution of S330/S331) were prepared by PCR and subcloned into the p3XFlag-CMV™-7.1 expression vector (Sigma-Aldrich) using Muta-Direct Site-Directed Mutagenesis Kit (#15071; Intron, Seongnam, Gyeonggi, South Korea). The mutations were confirmed by DNA sequence analyses (Bionics, South Korea). pSG5-HA-RIPK1 was kindly provided by Dr. Jin-Hyun Ahn (Sungkyunkwan University School of Medicine, Suwon, South Korea). For transient overexpression, cells were transfected using Omicsfect (OmicsBio, Taipei, Taiwan) in a serum-free medium for 24–48 hours, based on the manufacturer’s instructions.

### 4.3 Erythrocyte isolation and cell counting

For erythrocyte isolation, whole blood was obtained from mice in citrate-phosphate-dextrose buffer (16 mM citric acid, 90 mM sodium citrate, 16 mM NaH_2_PO_4_, 142 mM dextrose, pH 7.4) in a ratio of 1/10 of the blood volume. Then, the mixture was transferred to a fresh 1.5-ml Eppendorf tube. After gentle pipetting, the samples were centrifugated at 200×g for 20 min at room temperature. After removing the top and middle layers, 90% of the volume of the bottom layer was transferred to a fresh 1.5-ml Eppendorf tube. For washing, 1 ml of cold 1X phosphate-buffered saline (PBS; Gibco, #10010-023, USA) was added, mixed gently, and centrifuged at 2000×g for 20 min. The supernatant was discarded and the wash process was repeated three times. The erythrocytes isolated were prepared for the next experiment. For erythrocyte cell counting, the Scepter 2.0 Cell Counter (Millipore, #PHCC00000) and Scepter Sensors (Millipore, #MER-PHCC60050) were used.

### 4.4 Immunoprecipitation and western blot analyses

For Flag IP, cell lysates were incubated with agarose-conjugated anti-FLAG antibody (MBL, Woburn, USA) for 2 h at 4°C. For RIPK1, RIPK3, and HA IP, anti-RIPK1 (BD Biosciences, #610459, USA), anti-RIPK3 (Novos, #NBP1-77299, USA), anti-HA (Santa Cruz, #sc-7392, Dallas, TX, USA), antibodies were incubated overnight (O/N) at 4°C and then incubated with agarose-conjugated protein A/G (Santa Cruz) for 3 hours at room temperature (RT), respectively. As an immunoprecipitation (IP) control, control mIgG (R&D Systems, #MAB002) or control rabbit IgG (R&D systems, #AB-105-C) antibody was used. Purified proteins in the IP precipitates were washed three times with the wash buffer (150 mM NaCl, 2 mM EGTA, 2 mM MgCl2, 20 mM HEPES, pH 7.4, and 0.1% NP-40) and eluted with 4X sodium dodecyl sulfate (SDS) loading buffer at 95°C for 5 min. The eluents were analyzed by western blot with specific antibodies.

Western blot was performed as described previously (20). Briefly, cells were lysed with RIPA buffer (150 mM NaCl, 25 mM Tris–HCl (pH7.6), 1% sodium deoxycholate, 0.1% SDS, and 1% NP-40) (Thermo Fisher Scientific, #89901, Waltham, MA, USA) or 1% NP-40 lysis buffer (150 mM NaCl, 1 mM EDTA, 50 mM Tris–HCl (pH7.4), and 1% NP-40) supplemented with a protease inhibitor cocktail (Roche, Mannheim, Germany) and a phosphatase inhibitor cocktail (Roche). Total erythrocyte lysate (50–80μg) or total cell lysate (20–30μg) was loaded onto 8%–10% SDS-polyacrylamide electrophoresis gels. After antibody incubation, the EZ-Western kit (DoGenBio) or SuperSignal West Femto Chemiluminescent Substrate (Thermo Fisher Scientific, Inc.) and Amersham Imager 600 (GE Healthcare Life Sciences, Little Chalfont, UK) were used for signal detection. To quantify total O-GlcNAc signals, the immunoreactive whole lane band was detected and the integrated signal intensity was measured using AI600 imager system software. Thereafter, O-GlcNAcylation levels were normalized to the total protein levels and the amount of CBB staining using AI600 imager system software.

The antibodies used for western blotting or IP included anti-FLAG (MBL, #PM020), anti-GAPDH (Santa Cruz, #sc-32233), anti-HA (Santa Cruz, #sc-7392 for IP), anti-HA (Cell Signaling, #3724s for WB), anti-K48 (Cell Signaling, #8081s), anti-K63 (Cell Signaling, #5621s), anti-MLKL (Abcepta, #AP14272B), anti-mouse-phospho-S345-MLKL (Abcam, #ab196436), anti-O-GlcNAc (Thermo Fisher Scientific, #MA1-072), anti-PARP (Cell Signaling, #9532s), anti-Caspase 3 (Cell Signaling, #9662s), anti-Caspase 6 (Cell Signaling, #9762s), anti-Caspase 7 (Cell Signaling, #9492s), anti-Caspase 8 (Cell Signaling, #4927s), anti-RIPK1 (BD Biosciences, #610459 for IP), anti-RIPK1 (Cell Signaling, #34935s for WB), anti-mouse-phospho-S166-RIPK1 (Cell Signaling, #53286s), anti-RIPK3 (Novos, #NBP1-77299), anti-TRAF2 (Abcam, #ab244317), anti-cIAP1 (Enzo, #ALX-803-335-C100), anti-cIAP2 (R&D, #MAB817), anti-TAK1 (Cell Signaling, #4505s), anti-TAB1 (Cell Signaling, #3225s), anti-IKKβ (Cell Signaling, #8943s), and anti-NEMO (Abcam, #ab178872). Secondary antibodies included goat anti-rabbit IgG (#111-035-003, Jackson Laboratories, Bar Harbor, ME, USA), mouse anti-rabbit IgG (light-chain specific, #211-032-171, Jackson Laboratories), goat anti-mouse IgG (#115-035-003, Jackson Laboratories), and goat anti-mouse IgG (light chain specific, #115-035-174, Jackson Laboratories).

### 4.5 LDH release assay

The lactate dehydrogenase (LDH) Assay Kit-WST (Dojindo, #CK-12, Kumamoto, Japan) was used to assess LDH levels. Cells (3 × 10^4^) were plated into a 96-well cell culture plate. After drug treatment or DNA transfection, 100 μl of cell culture medium was incubated with the same volume of working solution and protected from light for 20 min. Then, 50 μl of stop solution was added and the absorbance at 490 nm was measured using a microplate reader. All LDH release analyses were performed in biological duplicates and are presented as the mean ± standard deviation.

### 4.6 Cell viability analyses

To determine cell viability, 1×10³ cells were plated in opaque-walled 96-well culture plates. The Cell Titer-Glo 2.0 assay reagent in an equal volume as the cell culture medium of TSZ (TNF + Smac– mimetic + Z–VAD–FMK)- stimulated cells or transiently transfected cells was added and mixed in an orbital shaker for 2 min (Cell Titer-Glo 2.0 assay Cell Viability Assay kit, Promega, G#9242, Madison, WI, USA). After incubation at RT for 10 min, luminescence was assessed with a Victor X5 multilabel plate reader (Perkin Elmer, Waltham, MA, USA). All cell viability assays were performed in biological duplicates and are presented as the mean ± standard deviation.

### 4.7 In-gel digestion

The eluate of RIPK1 was run on Bolt 4-12% Bis-Tris Plus Gels (Thermo Fisher Scientific). Briefly, protein bands were excised, destained, and washed. The proteins were reduced with 20 mM dithiothreitol and alkylated with 55 mM Iodoacetamide. After dehydration, the proteins were digested with 12.5 ng/μL Trypsin/Lys-C mix (Promega) in 50 mM ammonium bicarbonate overnight at 37°C. The resulting peptides were extracted from the gel serially with 10% formic acid (FA), 50% (v/v) acetonitrile (ACN) in 0.1% FA, and 80% ACN in 0.1% FA, dried, and stored at −20°C.

### 4.8 LC-MS/MS analysis, database search, and functional analysis

The extracted peptides were resuspended in 0.1% FA (solvent A). The samples were subjected to LC-MS/MS analysis integrated with an Easy nanoLC 1200 system and an Orbitrap Fusion Lumos Tribrid mass spectrometer (Thermo Fisher Scientific, San Jose, CA, USA) in triplicate. Peptides were loaded onto a C18 trap column (Acclaim PepMap100, Thermo Fisher Scientific, 75 μm X 2 cm, 100 Å), and separated on the C18 analytical column (PepMap RSLC, Thermo Fisher Scientific, 75 μm X 50 cm, 100 Å) with a 70-min linear gradient from 5% to 38% solvent B (0.1% FA in ACN) at a flow rate of 300 nl/min. The spray voltage of the column was set to 1.9 kV and the heated capillary was 275°C. The Q-Exactive was administrated in data-dependent acquisition mode with an MS survey scan, followed by ten MS/MS scans of the most abundant ions. The full MS scan range was from 400 to 1400 m/z and dynamic exclusion was applied for 30 s. In the event that oxonium product ions (m/z 204.0867, 138.0545) were observed in the higher-energy collisional dissociation (HCD) spectra, EThcD with a user-defined charge-dependent reaction time with 15% or 17% HCD supplemental activation was performed in a subsequent scan on the same precursor ion selected for HCD. The EThcD MS/MS scans had a resolution of 30,000 with the AGC target set to 1e5. The maximum injection time was 120 ms. The collected MS/MS spectra were analyzed using the SEQUEST algorithm present in the Proteome Discoverer software (Thermo Fisher Scientific; version 2.3). A protein database search was performed using the Uniprot human database (Release 21_01, 194,237 entries). Full tryptic specificity and up to two missed cleavage sites were permitted. Mass tolerances for precursor and fragment ions were set to 10 ppm and 0.02 Da, respectively. The fixed modification included in the search was carbamidomethylation (Cys) and variable modifications were oxidation (Met) and HexNAc (Ser and Thr). The data were also searched against a decoy database and the results were used to calculate q-values of peptide–spectrum matches (PSMs) using the Fixed Value PSM Validator within the Proteome Discoverer. Peptide and protein results were filtered to a 1% FDR.

### 4.9 Statistical analyses

All data were analyzed as the mean ± standard deviation. Statistical analyses were performed using Student’s unpaired *t*-test for two groups and one-way analysis of variance (ANOVA) with Sidak’s multiple comparisons test for multiple groups. The log-rank test was used to compare differences in survival between groups. GraphPad Prism software (Ver. 9) was used to determine statistical significance among multiple studies. Statistical significance was considered at *P < 0.05; **P < 0.01; ***P < 0.001.

## Data availability statement

The data presented in the study are deposited in the PRIDE repository, accession number PXD042579.

## Ethics statement

The animal study was reviewed and approved by The Institutional Animal Care and Use Committees of the Laboratory Animal Research Center at Yonsei University.

## Author contributions

JS, WY, and JC conceived the ideas. WY and JC supervised the project. JS, WY, and JC wrote the manuscript. JS designed, performed, and analyzed most of the experiments. YK and SJ assisted with the mouse experiments and data analyses. HK, HJ, and EY identified RIPK1 *O*-GlcNAcylation sites using MS analyses. Y-hL and IS provided experimental material and reviewed the manuscript. All authors contributed to the article and approved the submitted version.
