# Supplementary material for: O-GlcNAcylation of RIPK1 rescues red blood cells from necroptosis
Source: Front Immunol. 2023 Jun 9;14:1160490. doi: 10.3389/fimmu.2023.1160490 (PMC10289004; doi:10.3389/fimmu.2023.1160490)
Supplement: Supplementary file 1 [file DataSheet_1.pdf]

Supplementary Material

O-GlcNAcylation of RIPK1 Rescues Red Blood Cells from Necroptosis

Junghwa Seo[1], Yeolhoe Kim[1,2], Suena Ji[1], Han Byeol Kim[3], Hyeryeon Jung[1,3], Eugene C. Yi[1,3], Yong-ho Lee[1,4], Injae Shin[1,5], Won Ho Yang[1,2*], Jin Won Cho[1,2*]

Correspondence: Jin Won Cho: chojw311@yonsei.ac.kr

Won Ho Yang: bionicwono@yonsei.ac.kr

1.1 Supplementary Figures

Supplementary Fig. 1

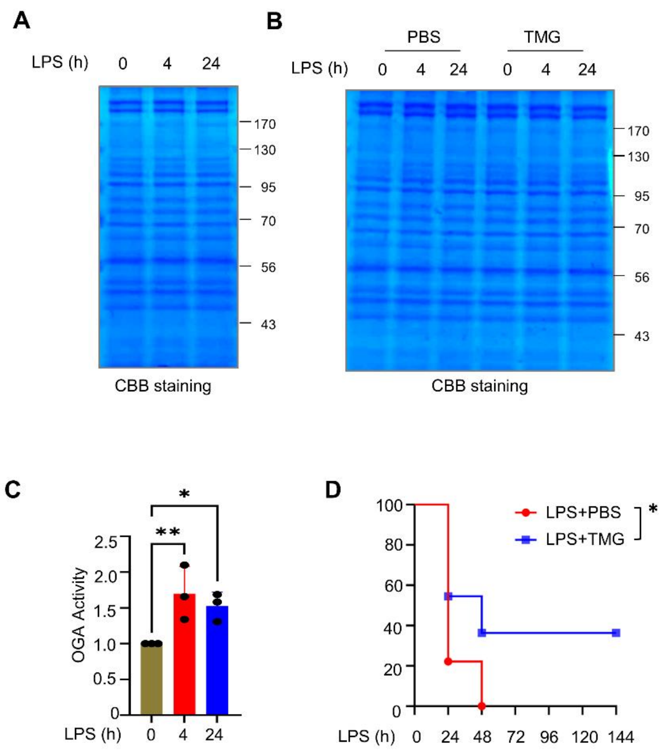

Supplementary Figure 1. OGA activity and lethality in LPS-injected mouse.

(A, B) Coomassie blue staining of total protein levels in lysates from Fig. 1A or Fig. 1B.

(C) Measurement of OGA activity of erythrocytes following LPS injection (**A**; one-way ANOVA with Sidak's multiple comparisons test; n=3)

(D) Survival measurement showing the effect of TMG administration on LPS-injected mice. Survival analysis is presented as a Kaplan–Meyer survival curve and was evaluated using a log-rank (Mantel-Cox) test; 20 mg kg^{-1} LPS, 40 mg kg^{-1} TMG, or the same volume of PBS (as a control) (LPS + PBS group [n]=9), (LPS + TMG group [n]=11). statistical significance was annotated as *P < 0.05, **P < 0.01.

Supplementary Fig. 2

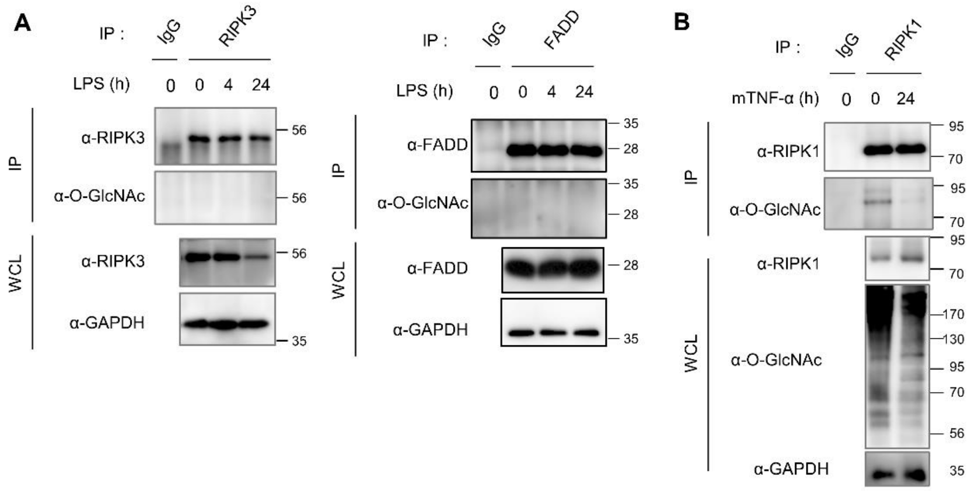

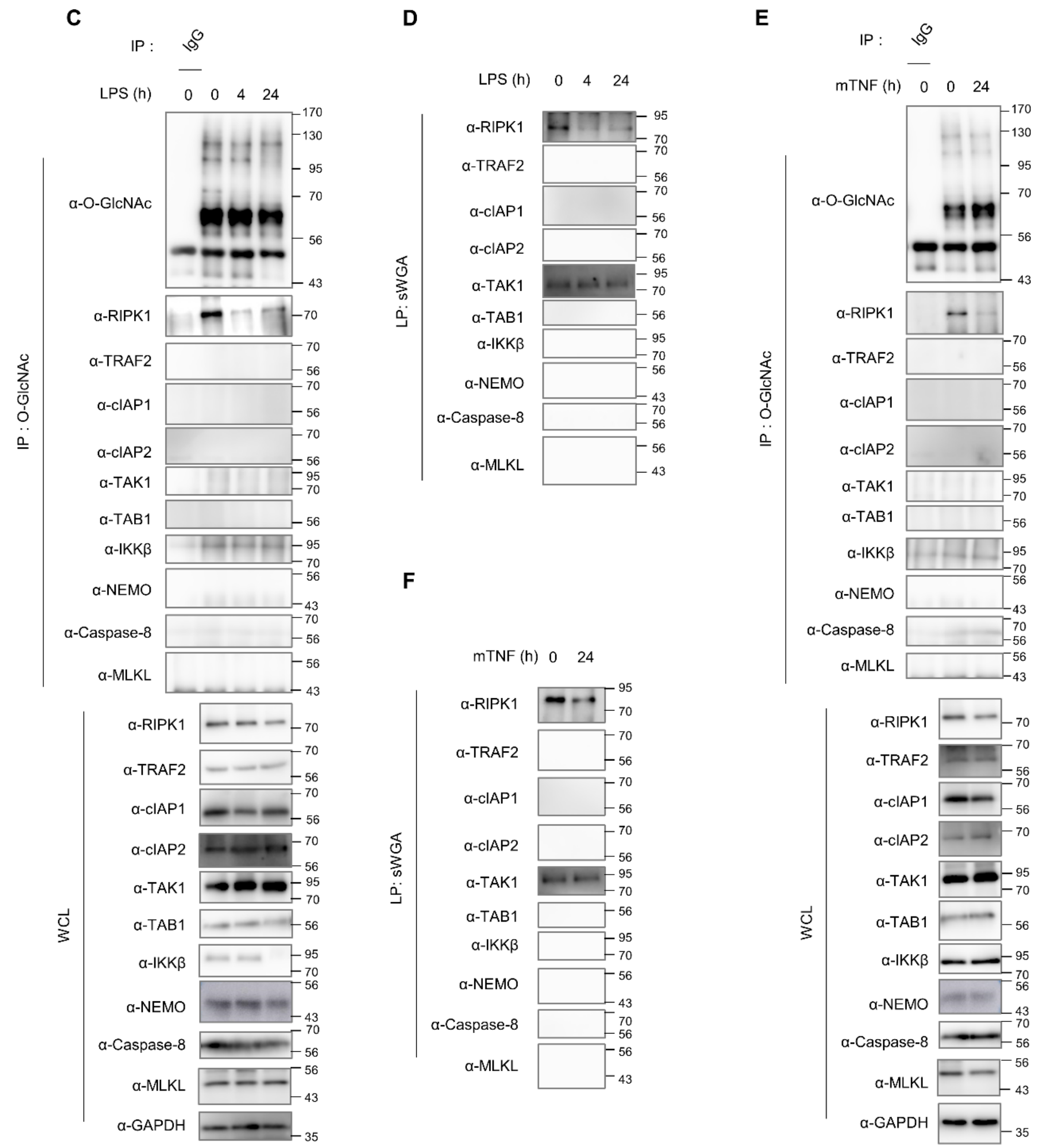
C
IP :
IgG
LPS (h)
0
0
4
24
IP : O-GlcNAc
α-O-GlcNAc
α-RIPK1
α-TRAF2
α-cIAP1
α-cIAP2
α-TAK1
α-TAB1
α-IKKβ
α-NEMO
α-Caspase-8
α-MLKL
WCL
α-RIPK1
α-TRAF2
α-cIAP1
α-cIAP2
α-TAK1
α-TAB1
α-IKKβ
α-NEMO
α-Caspase-8
α-MLKL
α-GAPDH
170
130
95
70
56
43
35
D
LPS (h)
0
4
24
LP: sWGA
α-RIPK1
α-TRAF2
α-cIAP1
α-cIAP2
α-TAK1
α-TAB1
α-IKKβ
α-NEMO
α-Caspase-8
α-MLKL
F
mTNF (h)
0
24
LP: sWGA
α-RIPK1
α-TRAF2
α-cIAP1
α-cIAP2
α-TAK1
α-TAB1
α-IKKβ
α-NEMO
α-Caspase-8
α-MLKL
E
IP :
IgG
mTNF (h)
0
0
24
IP : O-GlcNAc
α-O-GlcNAc
α-RIPK1
α-TRAF2
α-cIAP1
α-cIAP2
α-TAK1
α-TAB1
α-IKKβ
α-NEMO
α-Caspase-8
α-MLKL
WCL
α-RIPK1
α-TRAF2
α-cIAP1
α-cIAP2
α-TAK1
α-TAB1
α-IKKβ
α-NEMO
α-Caspase-8
α-MLKL
α-GAPDH

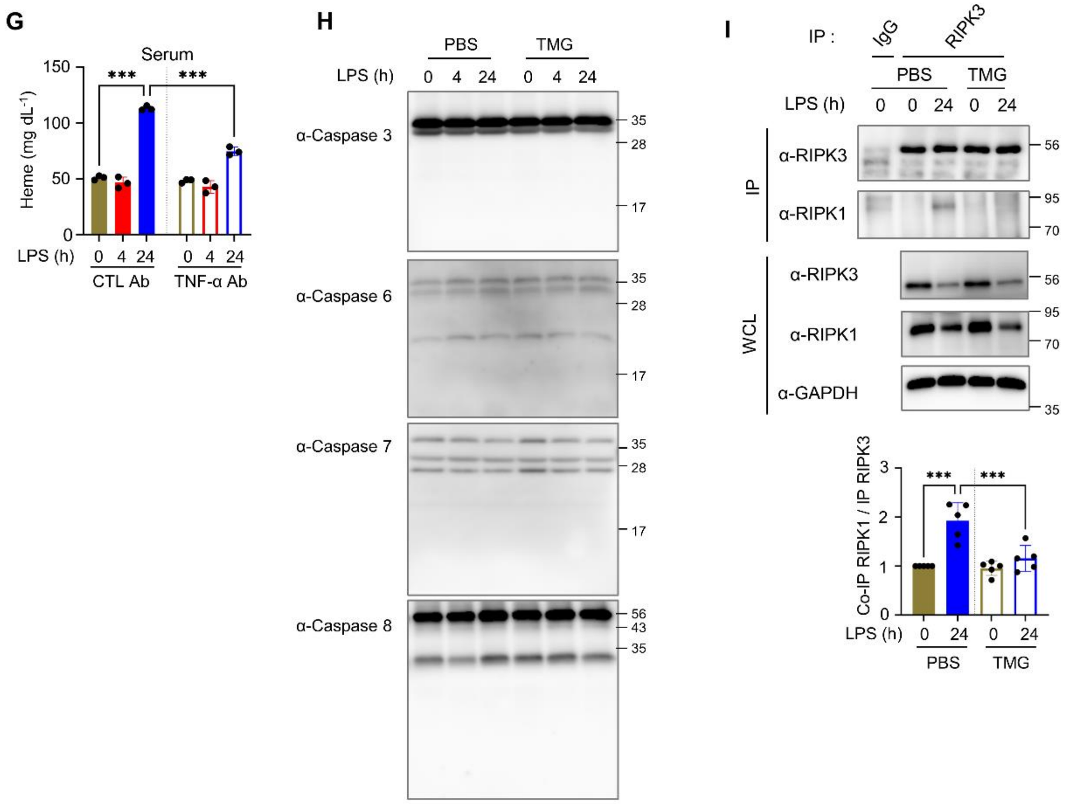

Supplementary Fig. 2. Investigation of *O*-GlcNAcylation and RIPK3-RIPK1 interaction of RIPK3 in erythrocytes.

(A) Immunoprecipitation (IP) assay to determine the *O*-GlcNAcylation of endogenous RIPK3 (left) and FADD (right) in erythrocytes isolated from LPS (10 mg kg^{-1} day^{-1})-injected mice during the indicated times. (A: the same amount of RIPK3 or FADD protein was immunoprecipitated from erythrocyte whole cell lysate.)

(B) Immunoprecipitation (IP) assay to determine the *O*-GlcNAcylation of endogenous RIPK1 in erythrocytes isolated from TNF-α (250 μg kg^{-1}) -injected mice.

(C, E) Immunoprecipitation (IP) assay to examine the *O*-GlcNAcylation of indicated proteins in erythrocytes isolated from LPS (10 mg kg^{-1} day^{-1}) or TNF-α (250 μg kg^{-1}) -injected mice using an O-GlcNAc antibody.

(D, F) Succinylated-wheat germ agglutinin (sWGA) assay to examine the *O*-GlcNAcylation of indicated proteins in erythrocytes isolated from LPS (10 mg kg^{-1} day^{-1}) or TNF-α (250 μg kg^{-1}) -injected mice.

(G) Heme assay to measure the concentration of free heme released into the serum. Serum was harvested at the indicated times after i.p. injection with 10 mg kg^{-1} day^{-1}LPS and 10 mg kg^{-1} day^{-1} TNF-α antibody or control antibody.

(H) Western blot analysis showing the levels of Caspase-3,6,7 and 8 after LPS injection in erythrocytes.

(I) Left: Co-IP assay showing the interaction between RIPK3 and RIPK1 in erythrocytes after a 24h LPS (10 mg kg^{-1} day^{-1}) injection. Right: graph showing the amount of co-immunoprecipitated RIPK1 normalized with immunoprecipitated RIPK3. (**G**, **I**; one-way ANOVA with Sidak's multiple comparisons tests; (G: n=3) (I: n=5). Data are presented as the mean ± standard deviation (SD); statistical significance was annotated as ***$P < 0.001$.

Supplementary Fig. 3

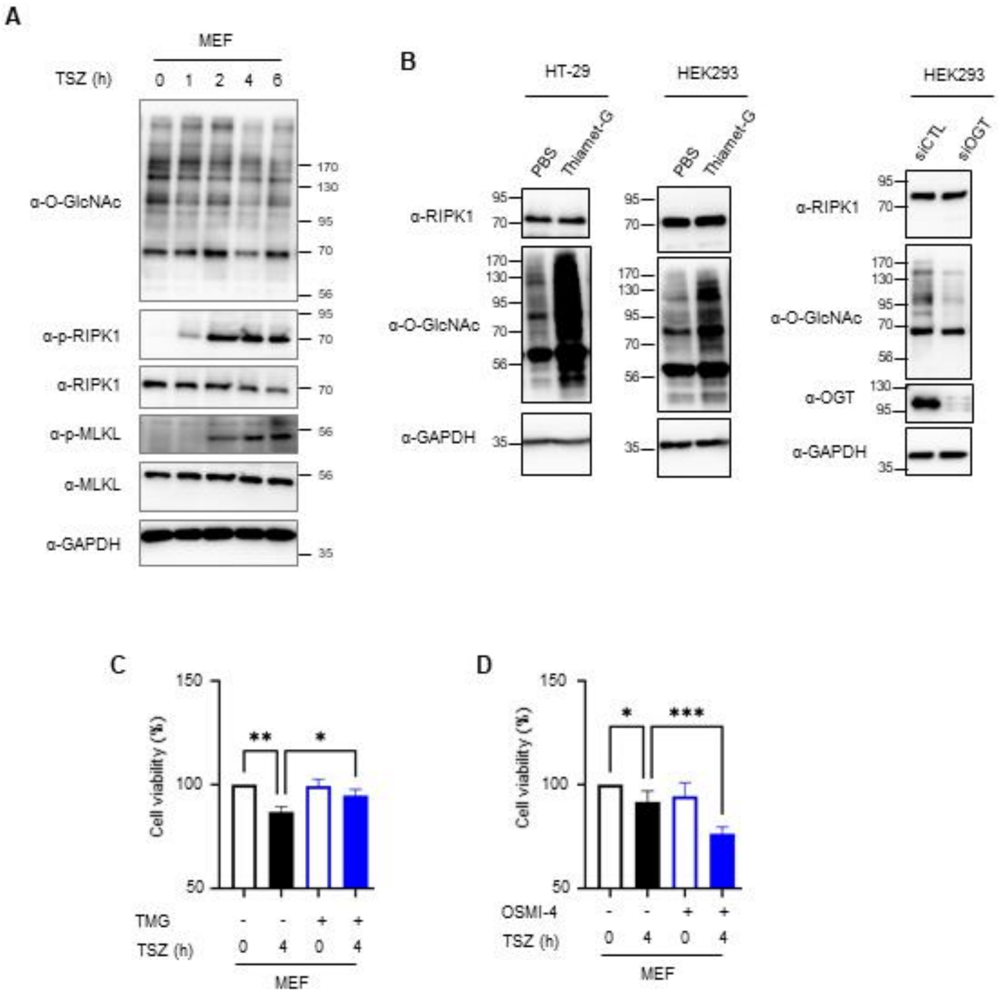

Supplementary Fig. 3. The decline of *O*-GlcNAcylation following TSZ stimulation and regulation of cell viability by TMG or OSMI-4 treatment in MEF cells.

(A) Western blot analysis showing the levels of *O*-GlcNAcylation after TSZ stimulation in MEF cells.

(B) Western blot analysis representing the RIPK1 protein levels upon altering *O*-GlcNAcylation status in HT-29 or HEK 293 cells.

(C, **D)** CellTiter-Glo assay to measure the cell viability of MEFs in necroptosis following TSZ stimulation. 1 μM TMG (C) or 2 μM OSMI-4 (D) were pretreated before a 4h TSZ stimulation. The y-axis starts at 50%. (**C**: n=3) (**D**: n=8)

(**C**, **D**; one-way ANOVA with Sidak's multiple comparisons test). Data are presented as the mean ± standard deviation (SD); statistical significance was annotated as *P < 0.05; **P < 0.01; ***P < 0.001.

Supplementary Fig. 4

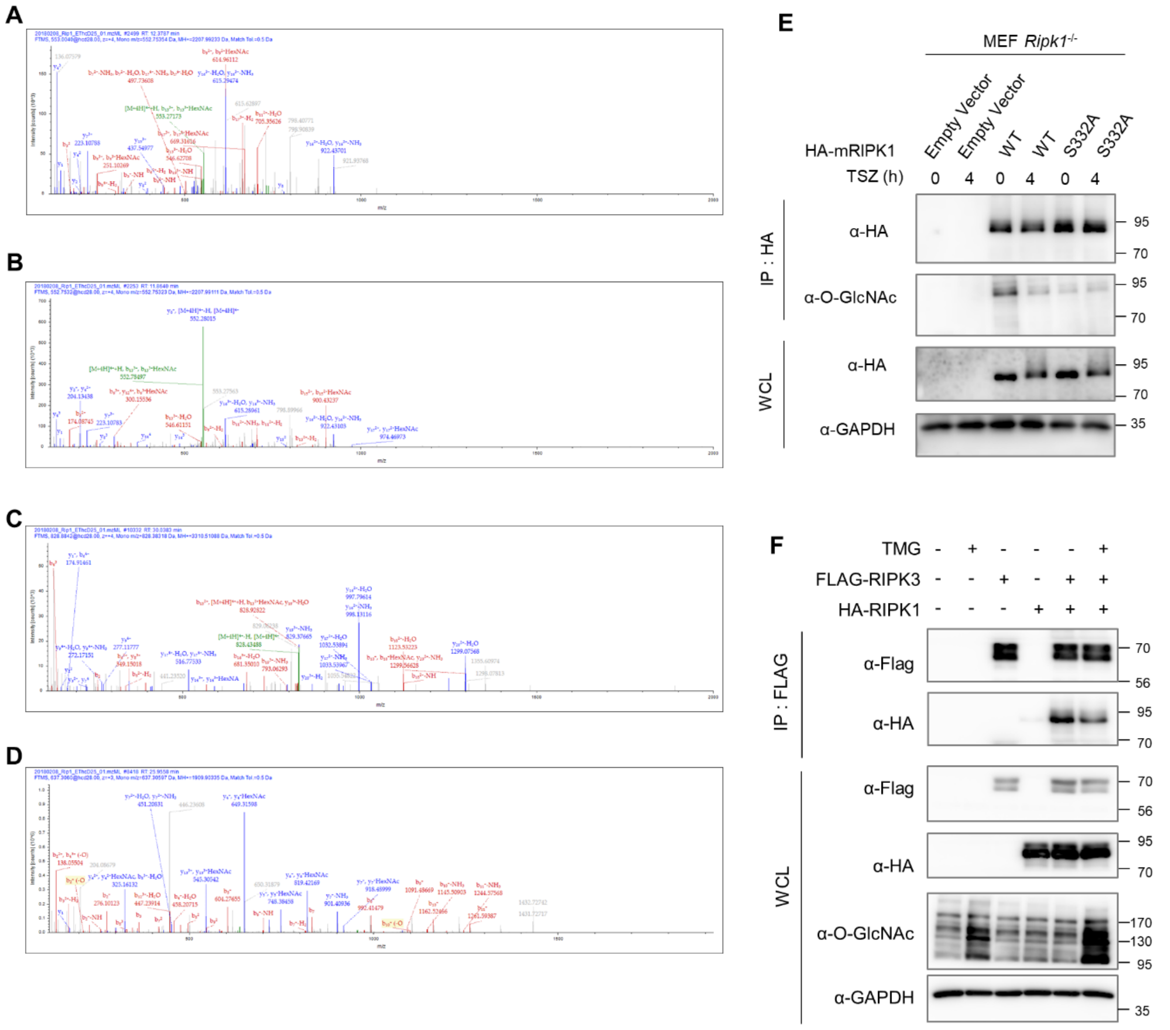

Supplementary Fig. 4. Mass spectrometry (MS) analysis to identify *O*-GlcNAcylation sites of RIPK1.

(A, **B**, **C**, **D)** Mass spectrometry (MS) analysis to identify *O*-GlcNAcylation sites in RIPK1. EThcD spectra of O-Glycopeptide **(A, B, C)** NVIKMKSSDFLESAG or **(D)** DCVAVPSSRSNSA from human RIPK1 is shown. The site of *O*-GlcNAc modification was identified as **(A)** serine 14, **(B)** serine 15, **(C)** serine 20, and **(D)** serine 330, although the moderate or low reliability. The b and y fragments detected are as indicated in the sequence.

(E) IP assay comparing *O*-GlcNAcylation levels between mouse WT RIPK1and mouse S332A RIPK1.

(F) Co-IP analysis showing that RIPK1-RIPK3 complex formation was attenuated by TMG in HEK293 cells. 10 µg of each DNA or empty vector (-) was overexpressed for 24h.

Supplementary Fig. 5

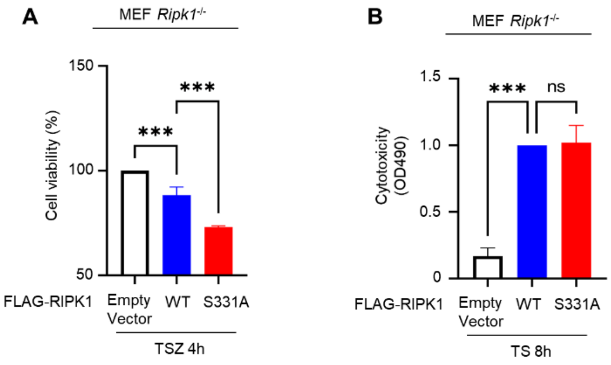

Supplementary Fig. 5. Measurement of cell viability in RIPK1 WT or RIPK1 S331A overexpressing MEF *Ripk1*$^{-/-}$ cells.

(A) CellTiter- Glo assay to measure the viability of MEF *Ripk1*$^{-/-}$ cells in necroptosis following TSZ stimulation. Before a 4h TSZ stimulation, 10 µg of each DNA construct was overexpressed for 20–24h. The y-axis starts at 50%. (one-way ANOVA with Sidak's multiple comparisons test, n=4).

(B) LDH release assay reflecting the cytotoxicity of MEF *Ripk1*$^{-/-}$ cells in apoptosis following an 8h TS stimulation. Before stimulation, 10 µg of each DNA construct was transfected. (one-way ANOVA with Sidak's multiple comparisons test, n=4).

Data are presented as the mean ± standard deviation (SD); statistical significance was annotated as ***P < 0.001.
